# Screening of ninety herbal products of commercial interest as potential ingredients for phytocosmetics

**DOI:** 10.1080/14756366.2020.1774571

**Published:** 2020-06-09

**Authors:** Mariacaterina Lianza, Manuela Mandrone, Ilaria Chiocchio, Paola Tomasi, Lorenzo Marincich, Ferruccio Poli

**Affiliations:** Department of Pharmacy and Biotechnology, University of Bologna, Bologna, Italy

**Keywords:** Herbal products, skin ageing, tyrosinase, elastase, phytocosmetics

## Abstract

Elastase and tyrosinase are important targets both for cosmetics and for dermatological disorders. In this work, ninety herbal products were tested as inhibitors of these two enzymes. Eleven extracts resulted strongly active. Four out of them (*Camellia sinensis*, *Ginkgo biloba*, *Rhodiola rosea*, *Vitis vinifera*) inhibited both enzymes, five (*Glycyrrhiza glabra*, *Ribes nigrum*, *Rheum officinale*, *Salvia officinalis*, *Tilia platyphyllos*) were active against tyrosinase only, and two (*Ceterach officinarum* and *Cinnamomum zeylanicum*) proved selectively active against elastase. The IC_50_ ranged from 3.1 to 104.9 μg/mL and 19.3 to 164.3 μg/mL, against elastase and tyrosinase, respectively. The most active extracts resulted enriched in flavonoids (from 1.47 to 56.47 mg RE/g of extract) and phenolics (from 37.43 to 123.56 mg GAE/g of extract), indicating also an antioxidant potential. Finally, a positive correlation between enzymatic bioactivities and phenolic content was also established.

## 1. Introduction

The demand for new skincare ingredients, especially based on natural products, strongly increased during the last few decades^1^^,^^2^. Plant bioactive metabolites are most of the time free from harmful side effects, hence, great importance is given to the research of naturally occurring anti-ageing agents. Several plants proved to be effective to slow down skin ageing^3^, acting as antioxidants, protecting skin against solar radiations^4^, and/or modulating the activity of enzymes involved in the ageing processes^5^, among which elastase (Ela) and tyrosinase (Tyr) are of remarkable importance.

Elastase activity increases significantly with age and after chronic UV-B irradiation ^6^, resulting in sagging due to loss of skin elasticity, thus inhibition of this enzyme is valuable strategy to slow down the intrinsic and extrinsic ageing processes^7^. Tyrosinase is responsible for skin hyper-pigmentation, as in case of melasma, freckles, ephelide and senile lentingines^8^ being an important target for skin-whitening agents.

This work aimed at identifying herbal products endowed with Tyr and/or Ela inhibitory activity (IA), thus potential anti-ageing agents. The bioactivity screening was carried out on extracts obtained from ninety commercial plants, widely used as ingredients for herbal preparations, including botanicals, herbal teas, and food supplements^9^^,^^10^. Considering the importance of polyphenols as antioxidant, the total phenolic and flavonoid content of the extracts was also determined and their content was also statistically correlated to the percentages of enzymatic inhibition.

## 2. Methods and materials

### 2.1. Plant material and extracts preparation

Plant samples were kindly supplied by Biokyma S.r.l., Anghiari (AR) Italy, and identified by Dr. Franco Maria Bini and vouchers of crude drugs were deposited in Department of Pharmacy and Biotechnology, University of Bologna (via Irnerio 42, Bologna, Italy) and reported in Table 1.

**Table 1.** Plants tested in this study, including their botanical name, family, organ/s used, voucher number, percentage of tyrosinase (Tyr IA %). and elastase inhibitory (Ela IA %) activity at 50 μg/mL, total phenolics (TPC) and flavonoids (TFC) content expressed in µg of gallic acid (GA) equivalent/mg of extract and µg of rutin (R) equivalent/mg of extract respectively.

Plant name	Family	Plant part	Voucher number	Tyr IA %	Ela IA %	TPC (µg AG eq/mg extract)	TFC (µg R eq/mg of extract)
*Ananas comosus* (L.) Merr.	Bromeliaceae	Stem	PU03555T	14	2	13.6 ± 0.8	0.8 ± 0.4
*Andrographis paniculata* (Burm. F.) Nees	Acanthaceae	Whole plant	PS16409T	14	0	60.3 ± 2.1	24.4 ± 2.2
*Angelica archangelica* L.	Apiaceae	Roots	PFU03844C	10	2	9.9 ± 0.3	0.4 ± 0.1
*Angelica sinensis* (Oliv.) Diels	Apiaceae	Roots	PU04044C	7	0	4.7 ± 0.1	0.7 ± 1.6
*Arctium lappa* L.	Compositae	Roots	PU06744C	24	7	35.0 ± 0.1	6.4 ± 0.3
*Arnica montana* L.	Compositae	Fruits	PZ05222I	24	8	49.5 ± 3.6	19.3 ± 0.3
*Artemisia dracunculus* L.	Compositae	Leaves	PZ19911T	16	6	60.4 ± 1.5	19.5 ± 2.3
*Astragalus propinquus* Schischkin	Leguminosae	Roots	PU06244C	17	6	9.8 ± 0.3	0.0 ± 0.0
*Avena sativa* L.	Poaceae	Aerials parts	PS06309T	8	14	25.6 ± 1.3	8.8 ± 0.2
*Berberis vulgaris* L.	Berberidaceae	Bark	PU07188T	13	2	30.5 ± 12.1	62.0 ± 3.6
*Boswelia sacra* Flueck	Burseraceae	Grains	PU30703I	19	0	1.4 ± 0.0	0.5 ± 0.7
*Camellia sinensis (L.)* Kuntze	Theaceae	Leaves	PU61111I	43	39	59.8 ± 3.7	26.2 ± 1.6
*Capsella bursa-pastoris* (L.) Medik	Brassicaceae	Flowered tops	PU08933T	8	0	40.8 ± 0.7	34.0 ± 1.6
*Capsicum annuum* L.	Solanaceae	Fruits	PU46555I	0	0	29.0 ± 2.2	7.0 ± 0.3
*Ceterach officinarum* Willd.	Aspleniaceae	Aerials parts	PFU59533T	24	63	84.7 ± 2.4	13.7 ± 0.5
*Cichorium intybus* L.	Compositae	Roots	BIOU15344C	0	0	6.8 ± 0.7	4.6 ± 0.2
*Cinchona succirubra* Pav.	Rubiaceae	Barks	PU15188C	23	16	82.9 ± 1.5	3.0 ± 0.1
*Cinnamomum zeylanicum* Blume	Lauracee	Barks	PU11188T	17	35	53.5 ± 1.5	5.3 ± 1.7
*Citrus aurantium* L. var. *dulcis* Hayne	Rutaceae	Flowers	PFU04922I	6	5	31.7 ± 0.7	14.2 ± 0.9
*Citrus aurantium* L. var. *dulcis* Hayne	Rutaceae	Zests	PFU04967T	8	11	21.4 ± 0.4	3.8 ± 0.4
*Coffea robusta* L.	Rubiaceae	Grains	PU09603C	23	22	100.9 ± 7.1	3.1 ± 0.0
*Coriandrum sativum* L.	Apiaceae	Fruits	PFU17955I	0	0	21.6 ± 0.5	3.1 ± 0.2
*Crataegus rhipidophylla* Gand.	Rosaceae	Flowers and leaves	PU08112T	23	17	77.9 ± 1.6	27.5 ± 2.5
*Cucurbita pepo* L.	Cucurbitaceae	Seeds	PU68277I	0	0	4.7 ± 0.3	0.2 ± 0.3
*Curcuma longa* L.	Zingiberaceae	Rhizomes	PU19344C	9	4	5.8 ± 0.1	3.3 ± 0.0
*Cynara scolymus* L.	Compositae	Leaves	PU11511T	22	19	83.7 ± 2.6	24.7 ± 0.5
*Cynodon dactylon* (L.) Pers.	Poaceae	Rhizomes	PU29499C	10	0	9.1 ± 0.2	0.9 ± 0.1
*Dioscorea villosa* L.	Dioscoreaceae	Roots	PS20044P	11	1	8.4 ± 0.2	0.0 ± 0.0
*Echinacea angustifolia* DC.	Compositae	Roots	PFZ20544C	14	21	22.2 ± 1.4	2.9 ± 0.2
*Echinacea pallida* (Nutt.) Nutt.	Compositae	Roots	PU20444C	7	0	21.5 ± 0.7	5.2 ± 0.4
*Echinacea purpurea* (L.) Moench.	Compositae	Roots	PU20644C	12	0	42.1 ± 0.6	11.9 ± 0.2
*Eleutherococcus senticosus* (Rupr. & Maxim) Maxim.	Araliaceae	Roots	PU21144C	10	8	31.5 ± 3.9	1.5 ± 0.1
*Elymus repens* (L.) Gould subsp. *repens*	Poaceae	Rhizomes	PU29999C	17	3	13.8 ± 0.5	0.3 ± 0.0
*Epilobium angustifolium* L.	Onagraceae	Flowered tops	PFU21933T	18	12	97.9 ± 0.9	22.5 ± 0.3
*Epilobium parviflorum* Schreb.	Onagraceae	Flowered tops	PU21833T	14	6	107.7 ± 1.5	22.6 ± 4.5
*Eschscholzia californica* Cham.	Papaveraceae	Whole flowered plant	BIOD3744I	19	13	31.6 ± 0.4	29.8 ± 0.4
*Foeniculum vulgare* Miller	Apiaceae	Fruits	PU25677I	14	0	37.3 ± 0.8	8.8 ± 0.3
*Fumaria officinalis* L.	Papaveraceae	Flowered tops	BIOF26733T	11	17	41.1 ± 3.7	17.6 ± 0.6
*Ginkgo biloba* L.	Ginkgoaceae	Leaves	PU28911T	38	34	79.8 ± 1.5	25.8 ± 1.0
*Glycyrrhiza glabra* L.	Leguminosae	Roots	PU34344C	59	2	37.4 ± 1.2	30.4 ± 2.0
*Handroanthus impetiginosus* (Mart. ex DC.) Mattos	Bignoniaceae	Barks	PU60244C	12	4	27.6 ± 1.6	2.7 ± 0.1
*Harpagophytum procumbens* DC.	Pedaliaceae	Roots	PU05544C	23	4	44.5 ± 0.0	2.4 ± 0.1
*Helichrysum italicum* G. Don	Compositae	Flowered tops	PU21333T	20	6	88.1 ± 0.8	27.8 ± 1.6
*Hibiscus sabdariffa* L.	Malvacee	Flowers	BIOU31922T	13	0	27.8 ± 0.6	4.4 ± 0.0
*Humulus lupulus* L.	Cannabaceae	Flowers	PS36722I	15	0	32.0 ± 0.5	6.3 ± 0.2
*Hyssopus officinalis* L.	Lamiaceae	Flowers and leaves	PFU31322I	18	3	69.9 ± 2.3	2.1 ± 0.4
*Ilex paraguariensis* A. St. Hil.	Aquifoliaceae	Flowered tops	PU38533T	20	0	113.8 ± 6.9	18.0 ± 0.2
*Lavandula angustifolia* Mill.	Lamiaceae	Flowers	PFU32722I	11	0	71.9 ± 3.4	6.9 ± 0.5
*Lepidium meyenii* Walp.	Brassicaceae	Roots	BIOZ37044P	9	0	8.3 ± 0.6	0.2 ± 0.0
*Malva sylvestris* L.	Malvaceae	Leaves	BIOU37311T	3	0	41.2 ± 1.9	26.8 ± 2.5
*Matricaria chamomilla* L.	Compositae	Flowers	PFU10522I	26	0	54.2 ± 3.8	36.4 ± 1.2
*Melissa officinalis* L.	Lamiaceae	Leaves	PU38911T	23	12	124.3 ± 7.6	7.5 ± 0.1
*Mentha* x *piperita* L.	Lamiaceae	Leaves	PU39511T	18	4	113.9 ± 9.1	60.5 ± 5.2
*Menyanthes trifoliata* L.	Menyanthaceae	Leaves	PU62711T	11	3	30.6 ± 2.2	13.3 ± 1.1
*Moringa oleifera* Lamk.	Moringaceae	Leaves	PU40711T	6	12	54.1 ± 0.9	22.6 ± 3.1
*Olea europaea* L.	Oleaceae	Leaves	BIOF42911T	9	0	76.5 ± 0.3	14.3 ± 1.4
*Orthosiphon aristatus* (Blume) Miq.	Lamiaceae	Leaves	PU43711T	12	7	51.7 ± 0.7	9.6 ± 1.4
*Panax ginseng* C. A. Mey.	Araliaceae	Roots	PC29144T	0	0	8.6 ± 0.2	0.1 ± 0.1
*Papaver rhoeas* L.	Papaveraceae	Petals	PFU44708T	15	10	75.1 ± 2.5	39.2 ± 1.3
*Paullinia sorbilis* Mart.	Sapindaceae	Seeds	PU30577I	22	6	51.1 ± 0.5	4.1 ± 0.1
*Peumus boldus* Molina	Monimiaceae	Leaves	BIOU08511T	17	2	88.1 ± 0.4	23.8 ± 0.5
*Piper nigrum* L.	Piperacee	Grains	PU46303I	3	10	35.4 ± 0.4	6.4 ± 0.0
*Plantago major* L.	Plantaginaceae	Leaves	PFU46711T	12	5	59.9 ± 0.8	10.6 ± 2.0
*Ptychopetalum olacoides* Benth.	Olacaceae	Wood	PU41104T	14	8	33.0 ± 0.7	2.6 ± 0.3
*Rheum officinale* Baill.	Polygonaceae	Rhizomes	PFU51799C	30	20	82.4 ± 2.9	16.1 ± 0.2
*Rhodiola rosea* L.	Crassulaceae	Roots	PU53244T	75	86	123.6 ± 18.6	1.5 ± 1.2
*Ribes nigrum* L.	Grossulariaceae	Leaves	BIOF52511T	48	19	96.4 ± 5.9	29.9 ± 0.8
*Rosa canina* L.	Rosaceae	Cinorrodes/seeds	PFU53310I	0	0	75.7 ± 1.1	1.6 ± 0.0
*Rubus idaeus* L.	Rosaceae	Leaves	PU54711T	7	10	127.2 ± 6.7	33.9 ± 1.5
*Salvia officinalis* L.	Lamiaceae	Leaves	BIOF56111T	43	9	95.9 ± 3.4	43.9 ± 1.0
*Sambucus nigra* L.	Adoxaceae	Flowers	BIOF56322I	19	9	80.1 ± 2.3	191.7 ± 2.2
*Satureja montana* L.	Lamiaceae	Leaves	PU56911I	21	0	84.2 ± 1.1	32.9 ± 0.2
*Schisandra chinensis* Turcz. Baill.	Schisandraceae	Fruits	PZ57455I	12	16	10.1 ± 0.4	0.7 ± 0.0
*Senna alexandrina* Mill.	Leguminosae	Leaves	PU58311T	12	21	31.9 ± 1.1	37.5 ± 0.1
*Serenoa repens* (W. Bratram) Small	Arecaceae	Fruits	PU58755T	12	17	25.7 ± 1.4	3.0 ± 0.2
*Silybum marianum* (L.) Gaert.	Compositae	Fruits	BIOU12155I	27	0	44.2 ± 2.4	2.3 ± 0.4
*Smilax aristolochiifolia* Mill.	Smilacaceae	Roots	PU55944C	0	0	20.4 ± 1.4	3.4 ± 0.5
*Theobroma cacao* L.	Malvaceae	Beans	PS18677I	24	15	91.3 ± 6.1	3.1 ± 0.1
*Tilia platyphyllos* Scop.	Malvaceae	Flowers and bracts	PU61533T	35	24	91.2 ± 1.3	13.9 ± 0.2
*Trifolium pratense* L.	Leguminosae	Fruits	PU62822I	22	4	69.8 ± 23.7	25.3 ± 0.1
*Trigonella foenum-graecum L.*	Leguminosae	Seeds	PU25377I	0	0	25.2 ± 1.1	15.3 ± 0.3
*Turnera diffusa* Willd. ex Schult.	Passifloraceae	Leaves	PZ19711T	17	13	58.7 ± 2.6	50.6 ± 0.7
*Uncaria tomentosa* (Willd. ex Schult.) DC.	Rubiaceae	barks	PU63288T	16	4	40.4 ± 0.5	2.6 ± 0.4
*Urtica dioica* L.	Urticaceae	Leaves	PU43911T	5	0	56.0 ± 1.4	16.7 ± 0.4
*Vaccinium myrtillus* L.	Ericaceae	Leaves	PU40511T	21	12	116.5 ± 2.0	43.6 ± 0.6
*Valeriana officinalis* L.	Caprifoliaceae	Roots	PU63744C	16	0	13.6 ± 0.5	2.2 ± 0.3
*Verbascum thapsus* L.	Scrophulariaceae	Fruits	PU64122I	7	0	9.7 ± 0.9	23.4 ± 1.7
*Viscum album* L.	Santalaceae	Leaves and twigs	PU65711I	10	9	21.9 ± 0.1	5.2 ± 0.1
*Vitex agnus castus* L.	Lamiaceae	Fruits	PU01355I	8	5	49.5 ± 1.2	22.9 ± 0.5
*Vitis vinifera* L.	Vitaceae	Leaves	PU66711T	42	36	81.1 ± 2.7	56.5 ± 1.5

Thirty mg of dried and powdered plant material were extracted by sonication for 30 min using 1.5 mL of EtOH/H_2_O (1:1). Crude extracts were obtained as reported by Chiocchio et al.^11^.

### 2.2. Enzyme inhibitory assays, total phenolic and flavonoid content

The assays were performed according to the methods described by Chiocchio et al.^11^, with slight modification for elastase inhibitory assay, where N-succinyl-Ala-Ala-Pro-Phe was used as substrate and p-phenylmethylsulfonyl fluoride (PMSF) from 1 to 250 μg/mL was used as positive control. For PMSF the assay was performed in 5% DMSO, thus, a proper negative control in the same conditions was used for the IC_50_ calculation.

The kinetic parameters for the enzymatic reactions in the assay conditions were K_M_ = 0.2 mM for both enzymes and V_max_ = 6 μmol/min for elastase and V_max_ = 10 μmol/min for tyrosinase.

The assays for phenolic and flavonoid contents were performed in Spectrophotometer (Jasco V-530) as described by Chiocchio et al.^11^.

### 2.3. Statistical analysis

Values were expressed as the mean ± SD of three independent experiments (each one performed in duplicate). Statistical analyses were performed using R Studio software (version 1.1.463) based on the R software version 3.5.2^12^.

Samples were compared by one-way analysis of variance (ANOVA) performed with “aov” function using “*stats*” package, followed by Tukey’s Honestly Difference (HSD) post-hoc test using TukeyHSD function presents in “*stats*” package^13^, considering significant difference at *p* values < 0.05. In order to determine the correlation between total phenolic and flavonoid content and enzymatic activities, Pearson correlation coefficient (*r*) was evaluated with “cor.test” function using “*stats*” package^14^.

## 3. Results and discussion

A first bioactivity screening was carried out on extracts at the fixed concentration of 50 μg/mL. The obtained results (Table 1) allowed the selection of eleven extracts, whose IA was higher than 30%, namely: *Camellia sinensis* Kuntze (leaves) (CCS), *Ceterach officinarum* D.C. (aerials parts) (COF), *Cinnamomum zeylanicum* Nees (barks) (CZE), *Ginkgo biloba* L. (leaves) (GBI), *Glycyrrhiza glabra* L.(roots) (GGL), *Rheum officinale* Baill. (rhizomes) (ROF), *Rhodiola rosea* L. (roots) (RRO), *Ribes nigrum* L. (leaves) (RNI), *Salvia officinalis* L. (leaves) (SOF), *Tilia platyphyllos* Scop. (aerial parts) (TPL) and *Vitis vinifera* L. (leaves) (VVI). Among them, four resulted active against both enzymes (CSI, GBI, RRO, VVI), five showed Tyr IA only (GGL, RNI, ROF, SOF, TPL), and two only Ela IA (COF, CZE).

**Figure 1.**
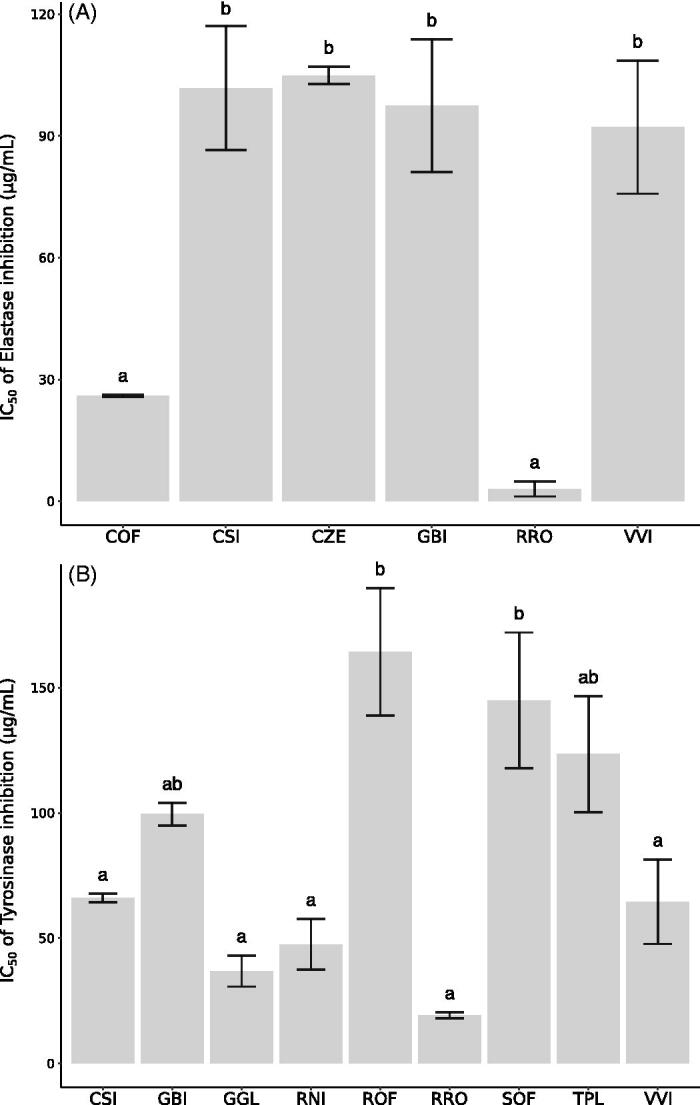
IC_50_ values of tyrosinase inhibition (A) and IC_50_ values of elastase inhibition (B) obtained for the most active extracts. Different letters within the same assay indicate significant differences in ANOVA test (*p* < 0.05). Results are expressed ad means ± SD of three independent experiments. COF: *Ceterach officinarum* DC.; CSI: *Camellia sinensis* Kuntze; CZE: *Cinnamomum zeylanicum* Nees; GBI: *Ginkgo biloba* L.; GGL: *Glycyrrhiza glabra* L.; RNI: *Ribes nigrum* L.; ROF: *Rheum officinale* Baill.; RRO: Rhodiola rosea L.; SOF: Salvia officinalis L.; TPL: *Tilia platyphyllos* Scop.; VVI: *Vitis vinifera* L.

The IC_50_ values of Ela IA of the six selected samples ranged from 3.1 ± 1.9 to 104.9 ± 2.1 μg/mL (Figure 1(A)), and among them, RRO and COF resulted the most potent elastase inhibitors (EI). These results are particularly promising, considering that the positive control (PMSF) used for Ela inhibitory assay showed IC_50_ of 42 μg/mL (241 μM). The extract of COF resulted the most potent Ela inhibitor of this screening, with an IC_50_ value of 26 ± 0.3 µg/mL. Regarding VVI, this study reported for the first time its activity against Ela, while its Tyr IA was already known^15^. Finally, this study provides new bioactivity data also for CZE, which is generally used as a food additive for its taste and scent. In particular, CZE expressed a selective Ela IA, showing an IC_50_ value of 104.9 ± 2.1 µg/mL, providing evidence in favour of the use of this herbal product also for skin care.

Concerning Tyr IA, the IC_50_ values calculated for the nine active extracts ranged from 19.3 ± 1.2 to 164.3 ± 25.5 μg/mL (Figure 1(B)), and the highest IA was shown by CSI, GGL, RNI, RRO, VVI.

Flowers and bracts of TPL are widely used in southern Europe folk medicine^16^, our results highlighted its strong Tyr IA, indicating a new potential use of this renowned herbal product as cosmetic ingredient. According to Chen et al.^17^, RRO acetone extract exerts Tyr IA with an IC_50_ of 181.8 ± 11.0 µg/mL, resulting remarkably less active then the hydroalcoholic extract tested in this study (IC_50_ of 19.26 ± 1.16 µg/mL). This difference may be due both to the different assay conditions, particularly incubation time and types of substrate, and to the diverse extraction method employed, since solvent properties strongly affect compounds extraction and consequently the inhibitory effect of a plant extract^18^^,^^19^. The same reasons could justify also the significant difference in Tyr IA found for CSI extract by Chen et al.^17^ (IC_50_ = 232.5 ± 3.3 μg/mL) and the one obtained in this study (IC_50_ = 66.04 ± 1.75 μg/mL).

Among the active principles contained in RRO tyrosol and salidroside are known to posses Tyr IA activity^20^. Regarding GGL, it is traditionally and commercially used for skin whitening formulations. The pyranoisoflavan glabridin showed promising anti-tyrosinase activity on melanoma cells and anti-melanogenesis activity on B16 murine melanoma cells. Additionally, reduced pigmentation and inflammation induced by UVB on guinea-pig skins at 0.5% w/v concentration^21^. Licuraside, isoliquiritin and licochalcone A also showed competitive inhibition on monophenolase activity of mushroom tyrosinase^22^, conversely, glabrene and isoliquiritigenin can inhibit both the reactions catalysed by tyrosinase^23^.

*C. sinensis* is widely used in skin care preparations for its peculiar catechines having significant antioxidant, anti-inflammatory and UV-protection activities. Moreover, tea polyphenols, i.e. (–)-epicatechin 3-*O*-gallate, (–)-gallocatechin 3-*O*-gallate, and (–)-epigallocatechin 3-*O*-gallate (EGCG) are known to possess tyrosinase inhibitory potential^24^.

Due to its popularity as cosmetic ingredient, CSI can be considered a further positive control in this screening. On this basis COF and RRO resulted very promising Ela inhibitors, being significantly stronger than CSI. While in the case of TI, GGL, RRO, VVI, and RNI showed an activity comparable to CSI.

Considering the antioxidant properties of polyphenols and flavonoids and their reported activity against several enzymes^25^^,^^26^, the total content of these classes of metabolites was evaluated in all the selected samples (Table 1). Pearson correlation test was performed to correlate the percentage of IA (showed by extracts at 50 µg/mL) to the phenolic and flavonoids content respectively.

A moderate positive correlation was found between the IA and the total phenolic content, the highest with TI (*r* = 0.4965449 and *p* values = 6.442e-07). A similar correlation trend was found in our previous work^11^, supporting the importance of phenolics in this biological activity. Conversely, for total flavonoid content no correlation was observed with both inhibitory activities. However, the eleven most active extracts resulted interestingly enriched in flavonoids (ranging from 1.47 to 56.47 mg RE/g of extract) and phenolics (ranging from 37.43 to 123.56 mg GAE/g of extract), which indicate also a potential activity as antioxidant agents.

## 5. Conclusions

Eleven extracts out of ninety resulted promisingly active against enzymes of cosmetic interest, showing IC_50_ values comparable or even lower than positive controls (PMSF and kojic acid). In particular, four out of them inhibited both enzymes, five tyrosinase only and two acted prominently only against elastase. For *C. officinarum* (aerial parts), *C. zeylanicum* (barks), *R.nigrum* (leaves), *T. platyphyllos* (flowers and bracts) and *V. vinifera* (leaves) the activity against one or both enzymes was reported for the first time, providing a new perspective for the use of these plants. Among them, *C. officinarum* (aerial parts) resulted one of the most potent EI, while *R. nigum* (leaves) and *V. vinifera* (leaves) showed the highest inhibitory activity against tyrosinase. The plants active against both enzymes (*C. sinensis*, *G. biloba*, *R. rosea*, *V. vinifera*) are potentially useful to develop cosmetics endowed with both skin-whitening and anti-wrinkles effect. The five plants active against tyrosinase only (*G. glabra*, *R. nigrum*, *R. officinalis*, *S. officinalis*, *T. platyphyllos*) are suitable for skin whitening agents, and the two active only against elastase (*C. officinarum* and *C. zeylanicum*) are interesting for selective anti-wrinkles cosmetics. Moreover, these plants resulted also enriched in polyphenols and flavonoids, conferring them additional antioxidant properties relevant for cosmetic ingredients and the total phenolic content showed a linear correlation with the enzymatic inhibitory activities.

Further biological and phytochemical studies are ongoing on the selected plants in order to identify the metabolites responsible for the observed biological activities.
